# Case Studies of Small-Medium Food Enterprises around the World: Major Constraints and Benefits from the Implementation of Food Safety Management Systems

**DOI:** 10.3390/foods12173218

**Published:** 2023-08-26

**Authors:** Jocelyn C. Lee, Marina Neonaki, Athanasios Alexopoulos, Theodoros Varzakas

**Affiliations:** 1 Independent Researcher, Benicia, CA 94510, USA; jlee@gourmetrail.com; 2 Department Food Science and Technology, University of the Peloponnese, GR24100 Kalamata, Greece; neonakimar@yahoo.gr; 3 Laboratory of Microbiology, Biotechnology and Hygiene, Department of Agricultural Development, Democritus University of Thrace, GR68200 Orestiada, Greece; alexopo@agro.duth.gr

**Keywords:** food safety management system (FSMS), Good Manufacturing Best Practices, prerequisite programs (PRPs), HACCP, systems thinking, food safety culture, food security, developing countries, developed countries

## Abstract

Global food safety and security are key principles to be followed in the context of the implementation of food safety management systems. The objective of this paper is to assess the contemporary developments of Food Safety Management System standards (FSMS) worldwide and to identify the primary constraints and advantages associated with their implementation by small and medium-sized enterprises across different regions. The effectiveness of these systems has also been evaluated. 116 case studies have been employed across developing and developed regions worldwide across 27 primary food sectors. After the implementation of FSMS, there was a significant increase in the percentage of companies that have implemented the international FSMS, both in developed (16.7% to 63.9%) and developing countries (26.6% to 48.1%). Certification has also increased from 34.2% to 59.6% in the total sample, namely from 33.3% to 61.1% in developed countries and from 34.6% to 59.0% in developing countries. There was a significant increase in medium vs. small company size (57.1% to 62.3%, *p* = 0.046), only in developing countries. Food safety culture and manager leadership implementation were increased to over 80% after FSMS implementation in both developed and developing countries (*p* < 0.001). Training, resources, and technology adequacy were also increased in all companies (*p* < 0.001).

## 1. Introduction

Food safety is an integral component of daily existence, and it is crucial to ensure its safety. Many nations lack appropriate microbiological criteria for food products, and even where they do exist, their enforcement is insufficient [1]. Hygiene is an essential component of food safety, yet consumer education regarding food hygiene is inadequate in many nations [2]. To ensure effective implementation and enforcement of food safety standards, governments should allocate adequate resources, strengthen their regulatory frameworks, and promote international cooperation and the exchange of information and best practices. Developing nations face numerous obstacles to the implementation of food safety standards, including inadequate infrastructure, limited financial resources, and a lack of understanding of food safety fundamentals. Recommendations include allocating adequate funds for the implementation and enforcement of food safety standards, strengthening regulatory frameworks, training personnel, educating consumers about food safety, and promoting international cooperation [3,4,5].

For preventing infectious diseases, promoting food safety, and ensuring the integrity of food products, food safety standards have become indispensable [6,7]. In developing nations, implementing food safety standards can reduce the incidence of infectious illnesses, improve the quality of life for the population, and provide access to international export markets. Implementing food safety standards in industrialized nations can reduce the incidence of infectious diseases, increase consumer confidence in food products, and facilitate international trade [1,2,4,5,8,9].

Even though many nations have made significant progress in implementing food standards, the results revealed both effective practices and obstacles in the implementation of food standards, as well as gaps in the implementation of HACCP and microbiological criteria. This study examined the global implementation of food safety standards with a focus on six crucial aspects: food standards, HACCP, prerequisites, microbiological criteria, food hygiene, and process controls. The findings revealed that many countries have implemented food standards, but implementation and enforcement differ. HACCP is widely recognized as a crucial aspect of food safety, but the lack of trained personnel to implement it is a significant obstacle. GMPs and GHPs are essential for ensuring food safety, but their enforcement in some nations requires improvement [10].

Microbiological standards are also essential for guaranteeing food safety, but many nations lack the necessary standards. Hygiene is an essential aspect of food safety, and process controls are required to guarantee the safety of food products. The implementation of food safety standards reduces the likelihood of infectious illness, improves product quality, boosts consumer confidence, and provides access to global markets. Governments were advised to allocate adequate funds for the implementation and enforcement of food safety standards, to strengthen regulatory frameworks, and to train qualified personnel. The food safety standards are a set of rules and guidelines designed to ensure the safety of food products for human consumption [6].

However, developing nations confront numerous obstacles, such as inadequate infrastructure, lack of water, polluted water, sanitation facilities, and financial resources. Food safety standards can be implemented in developing nations to reduce the incidence of foodborne illnesses, improve the quality of life for the population, and facilitate export market access [2,11]. However, developed nations are hindered by complex regulatory environments and limited resources. The implementation of food safety standards in developed nations can increase consumer confidence in food products, facilitate international trade, and reduce the incidence of infectious diseases [5,12,13,14,15,16].

This article presents the results of a global data analysis pertaining to the implementation of food standards, HACCP, prerequisites, microbiological criteria, food hygiene, and process controls. It was conducted to evaluate global food safety systems and to identify implementation flaws in food standards, HACCP, prerequisites, microbiological criteria, food hygiene, and process controls.

This study seeks to evaluate the global implementation of food safety standards and to identify the challenges and benefits of their implementation. It took nearly sixteen months of intensive research and analysis by a team of global food safety experts to produce a reliable, comprehensive, and inclusive article. The team performed a variety of tasks, including consulting with food safety specialists, designing, completing, compiling case study survey data, analyzing, and summarizing content. This article provides solutions and frameworks that can help policymakers, regulators, and industry stakeholders enhance the efficacy of food safety management systems.

Consequently, the objective of this paper is to assess the contemporary developments of Food Safety Management System standards (FSMS) worldwide and to identify the primary challenges and advantages associated with their implementation by small to medium-sized enterprises across different regions. The study aims to suggest potential ways to enhance or mitigate the impact of these systems in propelling countries forward. The effectiveness of food safety programs, including HACCP and food safety management systems, will also be evaluated. Lastly, the research seeks to establish a consensus on whether limitations of food safety management systems standards among small to medium-sized enterprises and their affordability may affect global safety and hinder the prevention of foodborne illnesses. This article provides solutions and frameworks that can help policymakers, regulators, and industry stakeholders enhance the efficacy of food safety management systems.

## 2. Food Safety Management Systems around the World

According to the WHO, foodborne illnesses affect a significant number of people globally, with approximately 600 million falling ill each year and resulting in 420,000 deaths [5,17]. Developing countries bear a higher burden of these illnesses and deaths due to challenges in managing food safety caused by limited infrastructure and resources. Food recalls are initiated when a product is deemed unsafe for consumption due to contamination or other hazards, and they can be initiated by food manufacturers, government agencies, or consumer complaints [4]. Developed countries have strict regulations and monitoring systems in place, such as the FDA in the US and EFSA in the EU, to ensure food safety. In contrast, developing countries may have less robust systems, leading to more frequent foodborne illnesses and recalls [12,13]. Recent examples of food recalls include salmonella contamination in pet treats, onions, and peaches in the US, listeria concerns in cheese and pork products in Europe, wheat flour contamination with metal pieces in India, and potential salmonella contamination in milk powder in Nigeria [18,19,20,21,22,23]. These examples emphasize the importance of strong food safety measures and monitoring systems worldwide to prevent and address contamination issues effectively.

Although there are several pillars and resources available to food processors (i.e., food safety legislation, policies, standards, and guidelines related to food safety systems implementation, controls and audits, worker training programs, etc.), foodborne illnesses continue to arise globally at an alarming frequency. As a result, this leads to expensive public health emergencies, product recalls and a reduction in consumer confidence in both food processors and brands [2,6,7].

A food safety management system refers to a methodical approach to managing and controlling food safety risks within a food establishment to ensure that food is safe for consumption. All businesses are obligated to establish, execute and sustain a food safety management system that is founded on the principles of Hazard Analysis Critical Control Point (HACCP) [6].

A food safety management system is a set of procedures designed to regulate food safety and guarantee that all food produced is of acceptable quality and safe for consumption. This entails outlining processes for each stage of the production process, from receiving supplies to delivering finished products. Furthermore, it is crucial that the food safety management system adheres to the principles of HACCP and each organization is responsible for developing procedures based on these principles. By law, food safety management systems are necessary to comply with food safety regulations.

Food standards are implemented to guarantee safety and quality in the food industry. They offer guidance to farmers and processors on how to handle food hygienically. These standards also establish the maximum levels of additives, contaminants, pesticides, and veterinary drug residues that are safe for consumption. Additionally, they specify the proper ways to measure, package, and transport food to ensure its safety. Food Standards play a crucial role in informing consumers about the nutritional content of food and allergen labeling, allowing them to make informed choices about what they consume [2,6].

**Figure 1:**
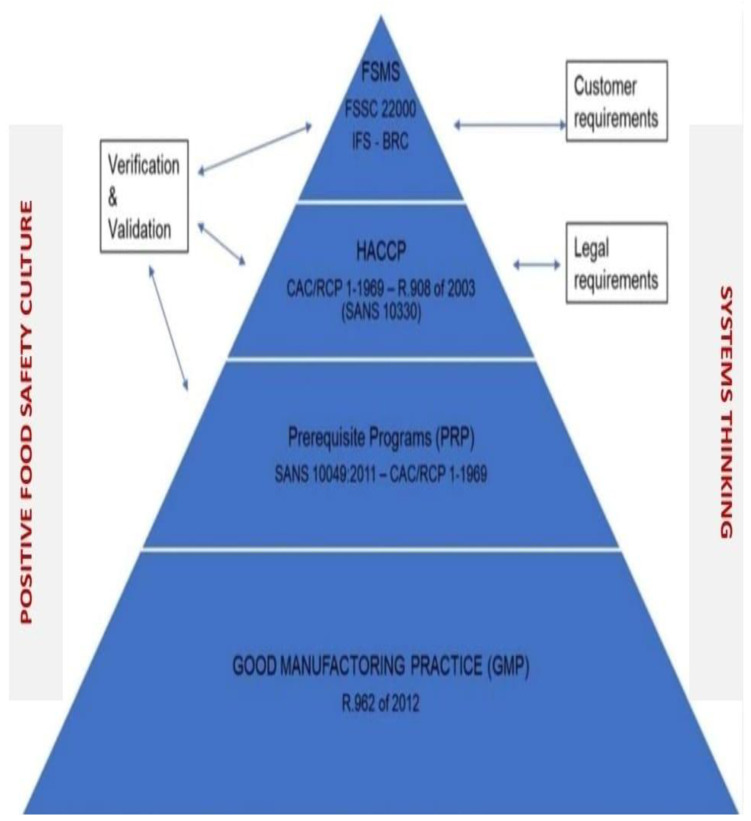
The FSMS Building Blocks and the Systems Thinking Approach (Adapted from “*What are Food Safety Management Systems?*”, https://chimerasystems.co.za/services/fsms/), accessed on 31 July 2022, Lee, J.C. et al. 2021 [6,27].

Food safety management systems (FSMS) have evolved to improve food safety practices and ensure the quality and safety of food products. The introduction of Hazard Analysis and Critical Control Points (HACCP) in the 1970s was a significant milestone, establishing preventive measures for identifying and controlling hazards. HACCP has become a global standard adopted by many countries. In the 2000s, the ISO 22000 standard [24] integrated HACCP principles with quality management systems, further enhancing food safety practices worldwide (Figure 1) [24,25,26,27].

Developed countries have well-established FSMS, regulatory frameworks, and training programs, enabling them to effectively control food safety risks and reduce foodborne illnesses. In contrast, developing countries face challenges due to limited resources, weak regulations, and low awareness of food safety. However, capacity-building initiatives aim to address these challenges and improve food safety practices in developing nations [23].

The impact of FSMS in reducing foodborne illnesses is evident in both developed and developing countries. However, more efforts are needed, especially in developing countries with greater food safety challenges, to ensure the effective implementation of good food safety practices and regulations.

### 2.1. Managerial Systematic Approach to Food Safety

Developing countries face challenges in implementing traceability programs due to limited resources, inadequate infrastructure, low literacy rates, and a lack of automation. This results in difficulties in tracking and tracing food products, which can increase the risk of foodborne illnesses. In contrast, developed countries have well-established traceability systems supported by advanced technologies and reliable infrastructure, enabling more effective implementation and comprehensive reporting [11,28].

Traceability programs in the food industry offer numerous benefits, including improved food safety, enhanced supply chain management, increased consumer confidence, compliance with government regulations, and reduced risks and wastage. These programs also create opportunities for value addition and improve market access for reliable food production intended for global export. However, the implementation of traceability programs entails certain drawbacks, such as higher operational costs, increased time requirements, and complexities associated with implementation and data management [29,30,31,32].

**Figure 2:**
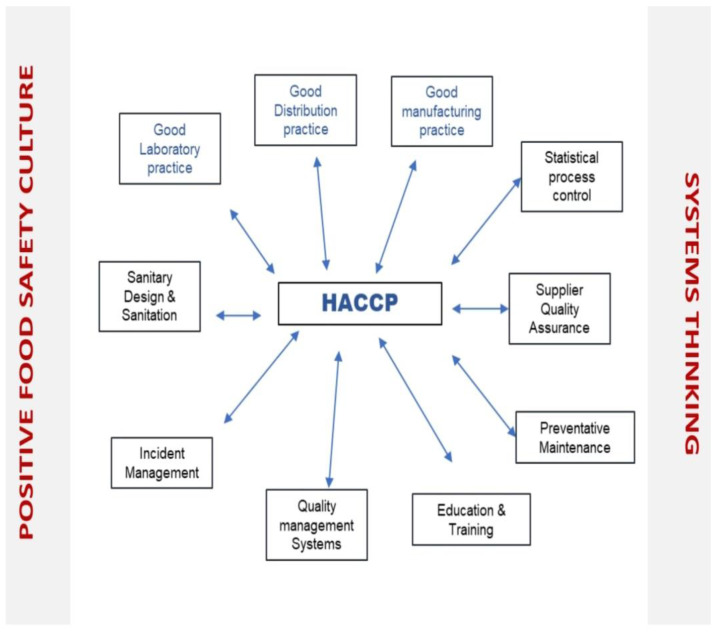
Managerial Systematic Approach to Food Safety Using HACCP and PRPs (Adapted from What is a Management System? Available online: https://images.app.goo.gl/Aqez5sSfpbvjQqmK7 (accessed 20 June 2021). Lee, J.C. et al. 2021 [6,33].

HACCP serves to establish, execute, and confirm the effectiveness of a Food Safety Management System. The fundamental basis for a practical and functional HACCP program in the prevention of foodborne illness outbreaks lies in the implementation of PRPs. A managerial approach that considers the entire system is essential when developing, implementing, and verifying HACCPs and PRPs. (Figure 2).

#### 2.1.1. Prerequisites: FSMS Foundation in Developing and Developed Countries

**Figure 3:**
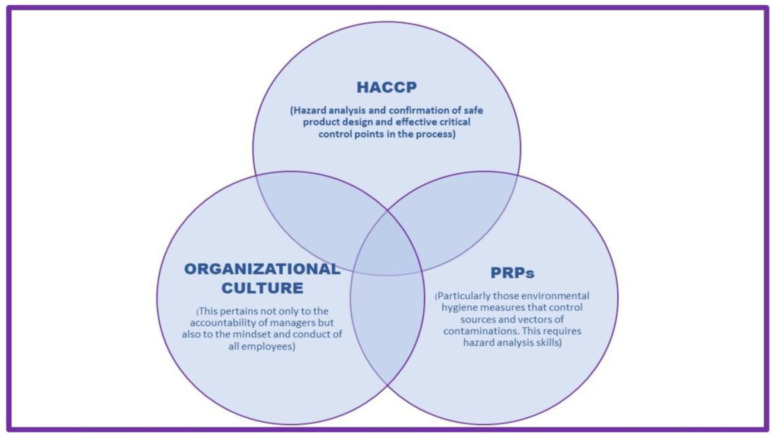
Organizational Food Safety Culture Works in Symbiosis with PRPs and HACCP (Adapted from: C.A. Wallace, and S.E. Mortimore, 2016) [7,35].

Various studies highlight the significance of prerequisite programs as the foundation of HACCP-based food safety management systems in both developing and developed countries. These programs play a crucial role in preventing food contamination throughout the entire food production process, encompassing aspects such as food handling, storage, hygiene, sanitation, pest control, training, and maintenance (Figure 3) [6,7,34]. Moreover, organizational Food Safety Culture Works in Symbiosis with PRPs and HACCPs.

Developed countries, such as the United States and the European Union, have established guidelines and regulations for prerequisite programs through regulatory agencies like the FDA and EFSA. In contrast, implementing and complying with these programs may pose challenges in developing countries due to limited resources, cultural factors, and lack of knowledge and awareness.

Nevertheless, studies demonstrate that implementing prerequisite programs in developing countries can lead to significant improvements in food safety and a reduction in foodborne illnesses. For instance, a study conducted in Ghana revealed that the implementation of a food safety management system incorporating prerequisite programs resulted in a notable decrease in foodborne illnesses. Similarly, a study conducted in China demonstrated that the implementation of prerequisite programs in food processing plants contributed to a reduction in the prevalence of microbial contaminants in food products, thus mitigating the risk of foodborne illnesses [1,11,34].

Overall, prerequisite programs play a critical role in preventing food contamination and ensuring food safety. Their effectiveness in both developing and developed countries may vary depending on the extent of implementation and adherence to regulatory guidelines and standards.

#### 2.1.2. FSMS and Systems Thinking

**Figure 4:**
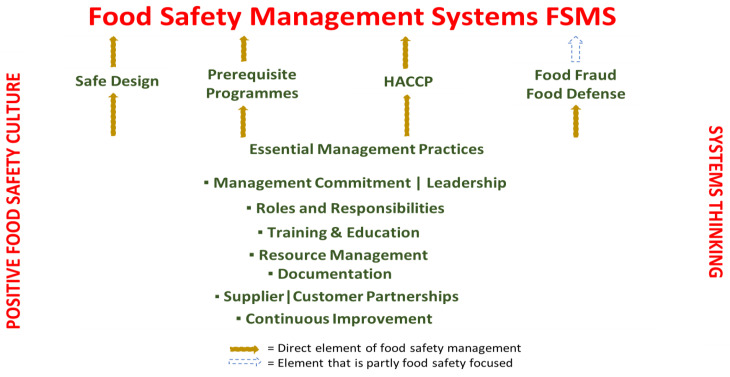
Organizational Food Safety Culture Works in Symbiosis with PRPs and HACCP (Adapted from: C.A. Wallace, W.H. Sperber, and S.E. Mortimore, 2018), Lee, J.C. et al. 2021 [6,36].

Managerial systems thinking involves taking a comprehensive approach to identifying the factors and interactions that contribute or could contribute to failures in a safety management system. In today’s world, many challenges are intricate and cannot be adequately addressed from a single viewpoint. Therefore, it is necessary from a single viewpoint. Therefore, it is necessary to examine the system as a whole and identify areas for restructuring. By combining systems thinking with food safety expertise and abilities, complex problems can be addressed effectively. Figure 4 illustrates the interaction between FSMS, necessary good management practices, positive food safety culture, and systems thinking [6,7].

#### 2.1.3. Positive Food Safety Culture

Food safety culture promotes food product and market safety in the food sector. Food safety culture impacts both developed and developing countries, but economic conditions, regulatory guidelines, and consumer awareness vary.

Food safety legislation, enforcement procedures, and authorities to safeguard the public are normally in place in developed countries. However, poor infrastructure, poverty, weak food safety rules, and limited resources make it more difficult for developing nations to establish a food safety culture. Despite such constraints, food safety awareness in developing nations has led to the establishment of various efforts and programs to improve food safety culture.

This study on the food safety culture element of FSMS and awareness in developing countries found that food safety culture is low due to low awareness and poor knowledge of food safety, inadequate infrastructure and regulatory standards, challenges implementing food safety guidelines, and insufficient financial resources to develop a robust food safety culture. However, developed countries have built robust infrastructure, resilient regulations, and enforcement mechanisms to control food safety concerns and promote a food safety culture.

Moreover, UNDP, WFP, WHO, and FAO initiatives have promoted food safety culture in developing countries and improved food safety management systems. These programs train, advise, and fund developing nations to improve food safety [7,37,38,39,40,41].

#### 2.1.4. Food Safety, Food Security, and Food Sustainability

Food safety and food security are closely related concepts that impact the health and well-being of individuals worldwide. Food safety ensures that food is prepared and consumed without harm to consumers, while food security ensures access to sufficient, safe, and nutritious food that meets dietary needs and preferences. Factors influencing food safety and security include environmental conditions, agricultural practices, trade policies, food processing, storage, distribution, consumer behavior, and public health interventions. Food safety and food security are interdependent, with unsafe food causing diseases and decreased productivity. Developing and developed nations face challenges in food safety, with microbial contamination, pesticide residues, and aflatoxins being more prevalent in developing countries. To improve food safety and security, both developing and developed nations must adopt a comprehensive strategy that involves all food chain stakeholders [42,43,44,45,46].

Ensuring global food security and sustainability is a major challenge for both developing and developed countries. Food security focuses on providing access to food for all, while sustainability aims to meet current needs without compromising the ability to meet future needs. In developing countries, food insecurity is caused by factors like poverty, inadequate infrastructure, climate change, and political instability, leading to malnutrition and health issues. Developed countries face challenges such as food waste, unsustainable farming practices, and excessive resource use. Efforts to promote food sustainability include adopting sustainable agriculture practices, reducing food waste, and encouraging plant-based diets. To address food security, measures like providing food aid, supporting smallholder farming, and improving education and healthcare access are implemented. Achieving food security and sustainability requires a comprehensive approach tailored to the unique challenges of each country. It involves ensuring equal access to nutritious food, implementing sustainable agricultural practices, and reducing waste across the food system [47,48,49,50,51].

#### 2.1.5. Food Fraud and Food Defense

Food fraud and food defense are major challenges globally, affecting both developed and developing countries. Food fraud involves intentional adulteration or misrepresentation of food products for financial gain, while food defense focuses on safeguarding food products against deliberate contamination.

Developing countries face greater vulnerabilities due to inadequate regulatory frameworks, weak enforcement, and poor infrastructure. Examples include the sale of misbranded and adulterated food products in India and the 2008 melamine scandal in China, where contaminated milk powder caused public outrage and exposed flaws in their food safety system [52,53].

Developed countries also experience food fraud incidents, such as the European horsemeat scandal and the U.S. honey laundering case, emphasizing the need for robust preventive measures. Food defense is a concern in developed countries, as the food supply-chain infrastructure may be targeted by terrorists, posing a risk to national security [34].

Both developed and developing countries must address these challenges by implementing effective regulations, strengthening enforcement mechanisms, improving infrastructure, and enhancing surveillance and monitoring systems. These measures are crucial to ensure the authenticity and safety of food products [54,55].

#### 2.1.6. Rewording the Guidelines Related to Microbiological Standards

Academics and researchers generally concur that food quality can be viewed from two perspectives: objective and subjective. The objective aspect pertains to the physical characteristics of the product and involves quality control and food technology. On the other hand, the subjective aspect pertains to consumer’s assessments and evaluations of the perceived quality characteristics of the product. Although food quality is a multifaceted and intricate concept, it is intrinsically linked to food safety. Food safety is the most crucial factor in ensuring that consumers purchase products that meet their safety expectations, and it is therefore subject to regulation. To ensure the quality and safety of food products, it is crucial to have monitoring systems in place at critical stages of the productions process. The use of accurate and quick analysis methods is essential to ensure that the product meets quality and safety standards and complies with labeling requirements. The prompt detection of spoilage agents, including bacteria, pathogens, and other microbial contaminants during food production and processing, is essential to minimize spoilage and ensure a safe food supply.

If the test results fail to meet the required criteria, food producers must follow the measures outlined by regulations. When repeated testing results are consistently satisfactory, food production companies must take immediate action to prevent the occurrence of microbial hazards. Companies must ensure that their products meet the specific criteria set for each type of food, without exception, depending on their characteristics [6].

As an example, *Cronobacter* spp., an opportunistic bacterium, can be found in various food types. While powdered infant formula (PIF) is the primary source of *Cronobacter*-related illnesses [22,23], the bacteria have also been detected in other foods such as fruits, vegetables, cereals, spices, starches, dry foods, and meat products. Several studies have investigated the prevalence and molecular characteristics of *Cronobacter* in different food sources, including studies conducted in Korea on poultry and vegetable products (published in 2019) and on powdered infant formula (published in 2012), a study in China on spices (published in 2018), and a study in England and Wales on clinical cases of *Cronobacter sakazakii* in infants (published in 2016). These studies contribute to our understanding of the occurrence and potential risks associated with *Cronobacter* in various food items [56,57,58,59,60,61].

Table 1 captures the main microorganisms of concern, toxins, and metabolites found in specific food categories [6].

#### 2.1.7. Constraints and Benefits: Third-Party Food Safety Schemes

Globally, third-party food safety schemes are growing in popularity because they enable food businesses to demonstrate their commitment to food safety to customers, regulators, and other stakeholders. In implementing and complying with third-party food safety schemes, however, both developing and developed nations face challenges and opportunities.

Constraints: Implementing third-party food safety programs can be expensive, particularly for small-scale and low-income food businesses. Investing in infrastructure, training, audits, and certification may be necessary. Some third-party food safety schemes may have excessively stringent requirements, making it difficult for small and medium-sized businesses to comply.

Complexity: Third-party food safety schemes may have complex requirements, and a lack of technical knowledge among businesses may impede their implementation and compliance.

Access: Access to third-party food safety schemes may be limited in some regions due to transportation or geographical barriers, particularly in developing nations.

Accrediting Bodies: Businesses and regulatory authorities may have little assurance of quality and consistency if certification bodies in different nations lack transparency, trust, and harmonization.

Benefits: Improved food safety third-party food safety programs are designed to ensure that food products are safe and free of contaminants, providing customers with confidence. Third-party food safety schemes provide assurance to consumers regarding the safety and quality of the food products they consume. Compliance with third-party food safety programs can provide access to new markets and competitive advantages over non-compliant competitors.

Continuous Improvement: Third-party food safety programs encourage food businesses to continually improve their processes and performance to maintain certification.

Harmonization: Third-party food safety schemes can promote harmonization in food safety standards and provide a common language across borders, resulting in a positive approach to global food security, public health, and commerce [62,63,64,65,66,67,68].

## 3. Materials and Methods

### 3.1. Sample Size

The sample of this study consisted of 116 small and medium-sized companies from all over the world. Thirty-six (36) companies from developed countries and 80 (80) companies from developing countries.

### 3.2. Content of Analyses-Case Study Survey Data Collection by Global Food Safety Professionals 

The objective of this research is to examine the impact of food safety standards on the food industry in both developing and developed regions around the world. To achieve this, nearly 116 surveys were conducted across 27 primary food sectors. The surveys were designed to collect relevant data while ensuring complete confidentiality of the company information.

The surveys were divided into two categories—one that collected data before the implementation of food safety standards and the other that collected data after the implementation of food safety standards. This division allowed for a comparative analysis of the impact of food safety standards on the food industry. To ensure that the data collected was comprehensive and representative, the surveys were divided into developing regions and developed regions. Additionally, they were conducted across various food sectors to obtain a broad understanding of the impact of food safety standards in the food industry.

Overall, the surveys were carefully designed and conducted to gain valuable insights into the effects of food safety standards on the food industry. The analysis of this data will provide useful information for policymakers and stakeholders in developing and implementing future food safety standards (See Sample Case Studies Survey, Appendix A).

In addition to the surveys, our team of food safety professionals provided valuable insights on regional food safety standards. These expert opinions were instrumental in enhancing the study and providing a more comprehensive understanding of the impact of food safety standards on the global food industry (See Acknowledgements and Thanks Section).

It took nearly 16 months of intensive research and analysis by a team of active global food safety experts to produce a reliable comprehensive, and inclusive case study. The team performed a variety of tasks, including consulting with food safety specialists, designing, completing, compiling case study survey data, analyzing, and summarizing content. A dedicated share drive was opened for this research which only the key authors had overall access to. When food safety professionals forwarded their Case Study surveys, the lead author reviewed each survey and contacted the contributor to correct or clarify the survey data. All revised surveys with comments and replies are retained as authentic records. The lead author virtually collaborated with most of the team food safety professionals upon request. All the while, the confidentiality of company information was maintained.

The authors organized the data by food sector, developed or developing region, and divided the data into before and after implementation of the Food Safety Management System FSMS. The organized data were divided into data reports and then converted into illustrative bar graphs.

The topic contents of this FSMS study are presented in a similar order to the elements of Implementation of Food Safety Management Systems Case Study Surveys. See Appendix A, Sample Case Study Survey.

### 3.3. Case Studies’ Confidentiality Integrity 

Case Study Survey Disclosure:

Your case study contribution(s) data will be included within a collective statistical analysis to be part of our research paper “Global Implementation of Food Safety Management System Case Studies by Small-Medium Enterprises: Major Constraints and Benefits”.

Confidentiality will be maintained. Therefore, this survey/questionnaire does not ask for company name, city, etc.

We ask non-specific general questions to gather pertinent information to be able to analyze and present the collective data in an informative format.

*“The aim of the research paper is to evaluate the young history of Food Safety Management Systems Standards and its major constraints and benefits arising from its implementation by small to medium size enterprises around the world. How to better improve or alleviate the constraints and to catapult the success of world food safety best management practices programs, HACCP and food safety management systems. Finally, to arrive at a consensus as to whether the implementation of Food Safety Management Systems Standards in small to medium food enterprises has so far impacted global foodborne illness (for better or worse).”* See Supplementary materials Sample Case Study Survey M1.

The following topic sections focus on worldwide FSMS elements and practices; before and after implementation of FSMS results for Developing and Developed Regions by Food Sector Category and the constraints and benefits of results of the Global Case Study Surveys visa vi the specific data and input contributed by reliable Food Safety Professionals around the world.

**Figure 5:**
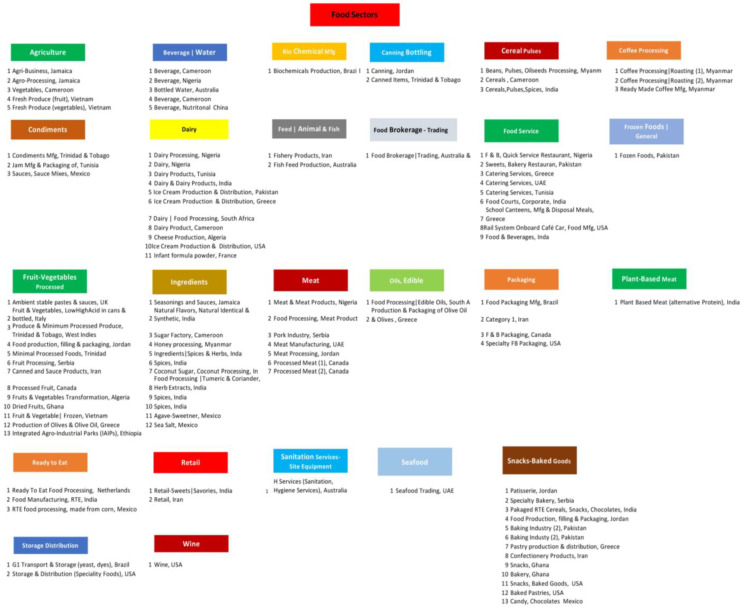
The Global Food Sector Chart illustrates the numerous food sectors represented in our data.

The Global Food Sector Chart illustrates the numerous food sectors represented by the Case Study Surveys, grouped by primary sectors for data analysis (Figure 5).

We followed the case study method “exploring a real-life, contemporary bounded system (a case) or multiple bounded systems (cases) over time, through detailed, in-depth data collection involving multiple sources of information as reported by Creswell [69] and Crowe et al. [70].

### 3.4. Analytical Framework

The processing of the statistical data was carried out with the statistical package IBM SPSS v.28. Scale reliability was examined with Cronbach’s alpha index.

Descriptive statistics are presented with absolute and relative frequencies (N, %) for categorical variables and median, interquartile range values for Likert scale variables. Dichotomous variables of FSMS Element Details before and after FSMS implementation were compared with the McNemar test, while Likert scale variables of attitudes towards constraints and incentives for FSMS implementation were compared with the Wilcoxon sign rank test [71].

## 4. Results and Discussion

In Table 2, the specific food sectors, and countries of the companies of the sample are presented. The most represented sectors in the sample were processed fruit and vegetables (13.8%), snacks and baked food (12.1%), dairy (9.5%), food service (9.5%), and ingredients (9.5%). Most companies were established in South Asia (22.4%), Africa (20.7%), East and West Asia (14.7%), Latin America and the Caribbean (13.8%), and Europe (12.1%).

### 4.1. Developed Countries vs. Developing Countries

**Figure 6:**
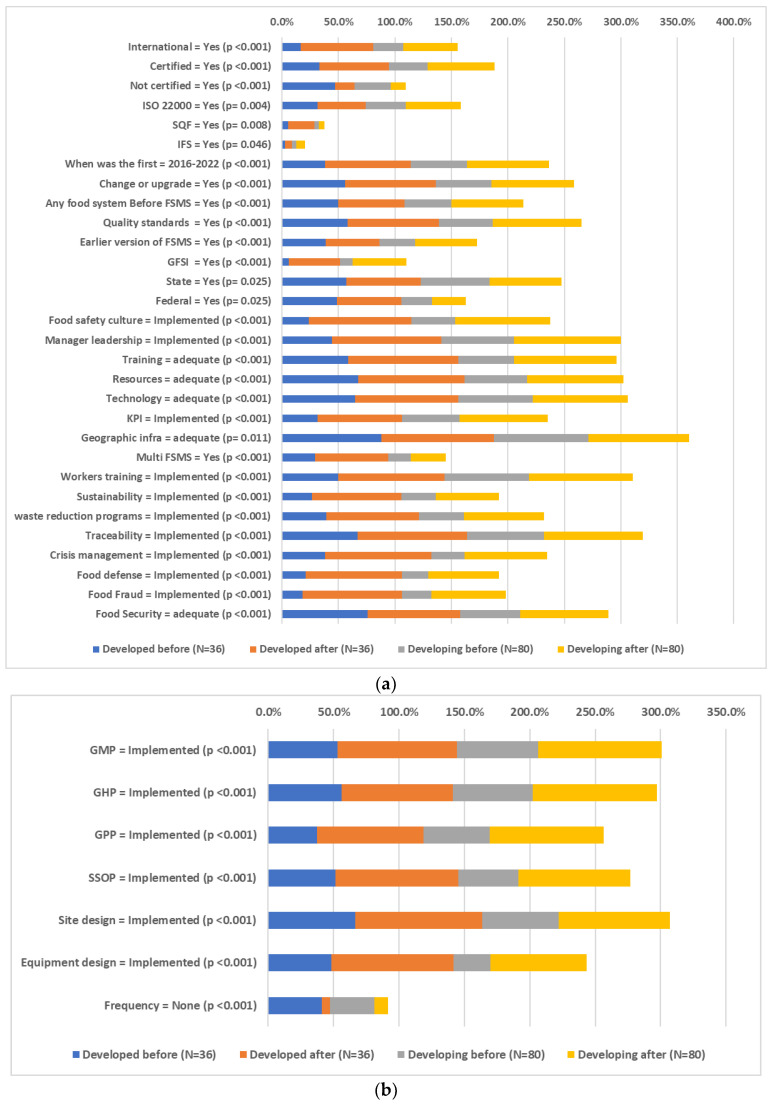

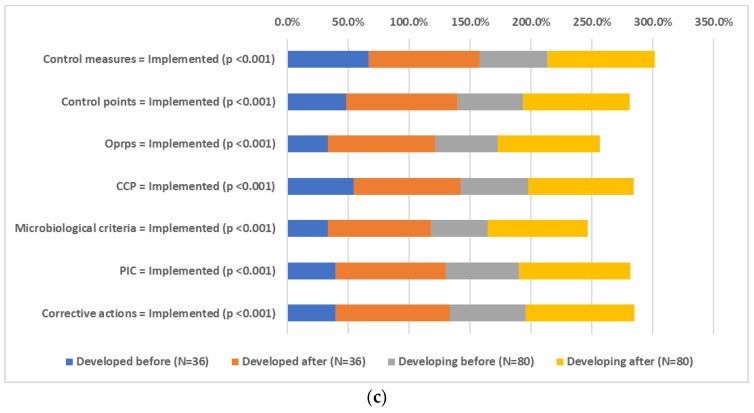
(**a**) Attitudes (FSMS element details) towards the constraints and benefits of implementing FSMS standards before and after implementation in developed and developing countries. Only attitudes with significant changes are presented. (**b**). Attitudes (Prerequisites) towards the constraints and benefits of implementing FSMS standards before and after implementation in developed and developing countries. Only attitudes with significant changes are presented. (**c**) Attitudes (HACCP) towards the constraints and benefits of implementing FSMS standards before and after implementation in developed and developing countries. Only attitudes with significant changes are presented.

Attitudes towards the constraints and benefits of implementing FSMS standards before and after implementation in developed and developing countries are presented in Figure 6a–c. After implementation, there was a significant increase in the percentage of companies that have implemented the international FSMS both in developed (16.7% to 63.9%) and developing countries (26.6% to 48.1%). Certification has also increased from 34.2% to 59.6% in the total sample, namely from 33.3% to 61.1% in developed countries and from 34.6% to 59.0% in developing countries. The increase in ISO 22000 implementation from 34.2% to 46.8% in the total sample presented a more significant increase for developing countries (35.5% to 48.7%, *p* = 0.008). Only developed countries presented a significant increase in SQF implementation (5.7% to 22.9%, *p* = 0.014) and only developing countries presented a significant increase in Food Standard Agency implementation (17.1% to 27.6%, *p* = 0.005). All companies have increased the rate of change or upgrade of FSMS standards (51.4% to 75.7%, *p* < 0.001). The implementation of quality standards increased from 51.4% to 78.9% (*p* < 0.001), the implementation of the earlier version of FSMS from 34.2% to 52.3% (*p* < 0.001) and the implementation of GFSI from 9.5% to 46.7% (*p* < 0.001). There was also an increase for all companies both in State (59.8% to 64.3%, *p* = 0.025) and Federal (33.9% to 38.4%, *p* = 0.025) Regulatory Agencies Inspections. There was a significant increase in medium vs. small company size (57.1% to 62.3%, *p* = 0.046), only in developing countries, while a smaller, non-significant shift was also detected in developed countries (41.2% to 44.1%, *p* > 0.05).

Food safety culture and manager leadership implementation were increased to over 80% after FSMS implementation in both developed and developing countries (*p* < 0.001). Training, resources, and technology adequacy were also increased in all companies (*p* < 0.001). Production yield adequacy remained at the same level (78.5% to 79.4%) before and after FSMS implementation. Findings are in agreement with the study of Nguyen et al. [72], who indicated that mandatory and voluntary regulations and standards play a pivotal role in global food chains by ensuring comprehensive, forward-looking strategies that prioritize risk management and ongoing enhancement within the Food Safety Management System (FSMS).

Key Performance Indicator (KPI), Multi-FSMS, Workers Training, Sustainability Programs, Food & Materials Waste, Reduction Programs, Lot Identification Traceability, Crisis Management, Food Defense TACCP Plan and Food Fraud VACCP Plan implementations also increased after FSMS implementation for all companies participating in the sample (*p* < 0.001). These results agree well with the study of Fotopoulos et al. [73], who reported that essential elements like a company’s characteristics (preliminary measures, machinery, and validation processes) and workforce attributes (staff availability, dedication, education, and willingness) are key factors when establishing an efficient HACCP system. As per the results, these underlying factors also greatly influence the system’s objectives concerning the recognition, evaluation, and management of food-related safety risks.

Attitudes towards food security in the region of the company’s activities were more positive after the implementation of FSMS in developing countries (53.1% to 78.1%, *p* < 0.001). Prerequisites implementations (GMP, GHP, GPP, SSOP, Site design, Equipment design) have increased by over 80% after FSMS implementation in all companies (*p* < 0.001), while internal audits implementations (annual, quarterly, monthly) have significantly increased to over 90% of the companies (*p* < 0.001). HACCP management system contents (control measures, control points, operational prerequisite, programs oPRPs, critical control points, processed based microbiological criteria and testing practices, monitor, verify, validate, record keeping, responsible person in charge PIC and deviations, corrective actions) have all significantly increased after FSMS implementation (*p* < 0.001). Our results agreed with those reported by Chen et al. [74], who indicated that the rate of defects in chaga products showed a noticeable decrease, and there was a significant reduction in the instances of process flow irregularities needing rectification (*p* < 0.05). These changes suggested an enhancement of safety and quality standards. With continued implementation over an extended duration, the advantages of the food safety management system would become even more pronounced, leading to substantial enhancements across a broader range of monitored aspects. Findings also agree with the study of Sun et al. [75], who indicated that once the prerequisite programs (PRPs) of Good Hygiene Practices (GHP) and Good Manufacturing Practices (GMP) are implemented, the Hazard Analysis and Critical Control Points (HACCP) system can be employed to control food safety risks. Moreover, the study by Cusato et al. [76] indicated the implementation of a food safety system in a dairy processing plant located in the State of São Paulo. After the implementation of the food safety system, a significant reduction in the yeast and mold count was observed (*p* < 0.05).

Relative to constraints, there was a decrease in companies reporting that FSMS implementation is an expensive and complicated task, i.e., there are economic, technological, and legislation constraints (*p* < 0.001), while there was an increase in companies reporting the non-familiarity of FSMS to customers and consumers (*p* < 0.001). In developing countries, the “lack of clarity” constraints decreased after FSMS implementation (*p* = 0.007).

There was a positive shift in attitudes towards the incentives of avoiding duplication between processes in developed countries (*p* = 0.013), improving quality of management in developing countries (*p* = 0.007) and providing evidence of legal compliance in both developed and developing countries (*p* = 0.033). Yet, after the implementation of FSMS companies in developing countries presented a more negative attitude towards the incentives of reducing product losses (*p* = 0.033), enhancing export competitiveness (*p* < 0.001), and improving company image (*p* < 0.001).

### 4.2. Differences per Geographical Region before and after FSMS Implementation

**Figure 7:**
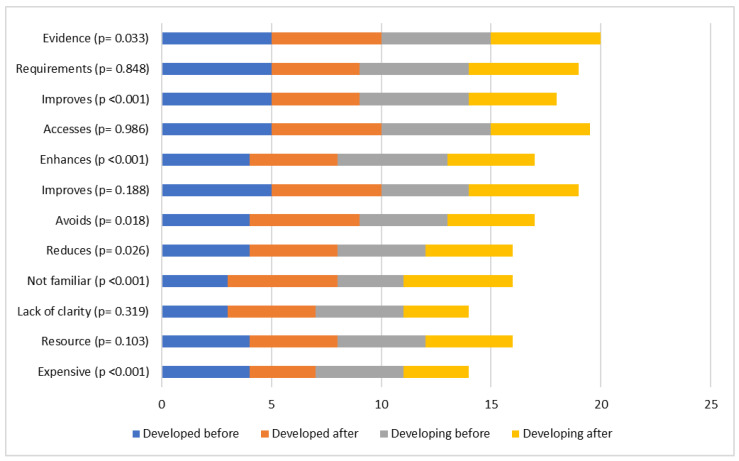
Median values of enterprise attitudes before and after implementation of FSM regarding specific constraints. Probability (*p*) values concern changes in attitude when both developed and developing countries are considered (Wilcoxon sign rank test, *p* < 0.01).

The differences before and after FSMS implementation for companies from different geographical regions of developed and developing countries are presented in Figure 7. UK and Australian companies did not present any significant shift in the application and attitudes toward FSMS standards; thus, they are not presented here. European companies reported significant increases in changes/upgrades of the FSMS standards, application of Quality standards, GFSI implementations, Food safety culture and manager leadership applications, multi-FSMS, Sustainability, waste reduction programs, crisis management, food defense, and food fraud systems integration. Also, significant increases in GMP, GHP and Equipment design prerequisites application, as well as all HACCP systems, were detected in European companies.

North American companies (US and Canada) indicated a significant increase in the implementation of International FSMS, Certification and SQF integration, changes which were not detected in Europe. Quality standards and GFSI integration were also increased for the North American sample, as well as Food safety culture, Manager leadership, Training, KPI, Workers’ training, Sustainability, Traceability and Crisis Management. Moreover, prerequisites and HACCP implementations have increased to 80–100% after FSMS implementation in US and Canadian companies.

African companies reported significant increases in certification, changes/upgrades of the FSMS standards, application of Quality standards, GFSI implementations, Food safety culture, and manager leadership applications, adequacy of Training and Resources, KPI implementation, Waste reduction programs, Traceability, Crisis management, food defense, and food fraud systems integration, as well as food security adequacy. Also, significant increases in prerequisites and HACCP systems application were reported in African companies. Similar trends were reported in Latin American countries.

For Indian companies, on the other hand, international FSMS application and certification have presented a significant increase, larger than the ones reported for African or Latin American companies and similar to the levels of companies from developed countries. Yet, Indian companies presented high rates of most prerequisites and HACCP applications, even before FSMS integration, and significant increases were detected in SSOP, Site design, Equipment design prerequisites as well as HACCP Microbiological criteria and PIC that had lower integration rates before FSMS implementation. Other companies in South Asia (Iran, Pakistan) presented similar differences to Indian companies, apart from HACCP integration which increased from 30–50% before to approximately 90% of companies after FSMS implementation.

Findings are aligned with the study by Grover et al. [77], who explored the use of quality management tools for identifying and prioritizing challenges faced by small food establishments during the adoption of preventive controls mandated by the FSMA legislation.

European private food safety standards exert considerable influence on global food and agricultural supply chains. They act as the link between consumer expectations and producers to bring the safety standards of exporting countries to par with, and sometimes well above, EU legislation, as reported by Rao et al. [78]. Private standards also allow the retail sector to govern agri-food supply chains from a distance, without taking on additional legal or financial obligations. The most observable effects of private food safety standards are characterized by changes in governance patterns, supply chain structures, access to international markets, and increasing demands with respect to non-safety attributes of food products.

Finally, East, and West Asian companies established in Myanmar, Vietnam, China, Jordan and UAE reported similar changes to Indian companies yet greater significant increases in KPI implementation, Geographic infrastructure adequacy, Multi FSMS, Workers’ training, Sustainability, Waste reduction programs, Traceability, Crisis management, Food Defense, and Food Fraud systems implementation. Such as in Iran and Pakistan, companies established in East and West Asia increased the integration of HACCP systems from 20–50% to 80% after FSMS implementation.

Attitudes towards constraints of FSMS implementation as an expensive and complicated task were reduced for North American, African, Latin American, and Asian companies (except for Iran and Pakistan). The constraint of non-familiarity of FSMS to customers and consumers increased for North American, European, African, Latin American, and Asian companies (except for Iran and Pakistan). For North American and African companies, the incentives of reduction in product loss and meeting customers’ requirements were decreased, while the incentive of providing evidence for legal compliance increased. European companies were the only geographical sector that increased the belief in avoiding duplication between processes. Companies from Latin America and India presented a positive shift in the belief that FSMS standards implementation improves the quality of management.

### 4.3. Differences per Sector before and after FSMS Implementation

**Figure 8:**
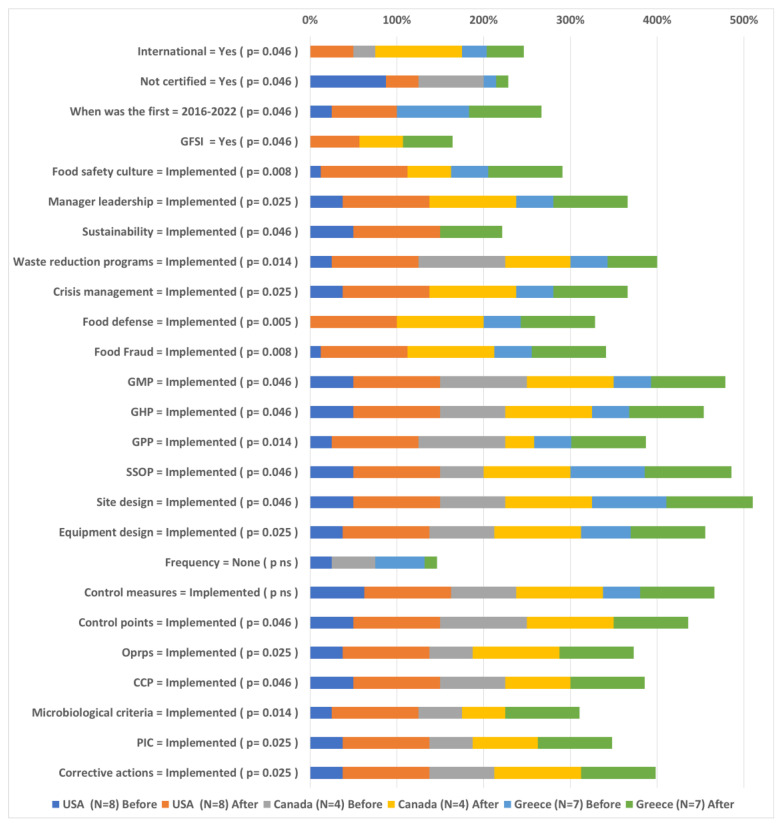
Differences per sector before and after FSMS implementation.

The processed fruit and vegetables, snacks and baked goods, and ingredients sectors presented a significant improvement in International FSMS implementation. The dairy, meat, and ingredients sectors expanded the rates of certifications. The snacks and baked goods and dairy sectors increased the rates of upgrades and changes in standards. Quality standards application has increased in the processed fruit and vegetables, snacks and baked goods, and dairy sectors, while GFSI implementation has increased also for ingredients and meat sectors. Moreover, all sectors presented significant increases in the implementation of Food safety culture, Manager leadership, and Training adequacy. Implementation of workers’ training, Sustainability, waste reduction programs, Traceability, Crisis Management, Food Defense, and Food Fraud systems has increased for all sectors as well. Significant large improvements were also observed in prerequisites and HACCP systems applications for sectors that presented lower integration before FSMS implementation, and smaller non-significant improvements have also been detected for the ingredients and meat sectors, even though prerequisites and HACCP integration was sufficient even before the FSMS (Figure 8).

No significant change in the incentives to implement FSMS Standards was detected within different sectors before and after FSMS implementation. The sector of snacks and baked goods, as well as the ingredients sector, perceived the FSMS after implementation as less expensive and complicated, compared to their attitudes before implementation. Yet, the sectors of processed fruit and vegetables, snacks and baked goods, food service, ingredients, and meat have reported greater constraints of non-familiarity with FSMS standards to consumers and customers after FSMS implementation. These findings well agree with the study of Wilcock et al. [79] who indicated that the main motivating factors for HACCP implementation were the likelihood of future regulation, the value of HACCP for marketing, and avoiding food safety problems. They also mentioned that successful implementation depended heavily on management commitment. They also used semi-structured in-depth interviews from different food sectors, including meat, wine, baked goods, flavors, and minimally processed fruits and vegetables. Finally, the implementation of HACCP led to increased food safety and product quality.

All indicators are very good as shown in Table 3 (>0.7). A low Cronbach’s alpha index appears for Constraints, although it is still marginally acceptable because it is >0.5.

### 4.4. Limitations of the Study

- Financial

Developing nations face numerous obstacles to the implementation of food safety standards, including inadequate infrastructure, limited financial resources, and a lack of understanding of food safety fundamentals. Recommendations include allocating adequate funds for the implementation and enforcement of food safety standards, strengthening regulatory frameworks, training personnel, educating consumers about food safety, and promoting international cooperation [3,4,5].

- Infrastructure

Developing nations confront inadequate infrastructure, lack of water, polluted water, sanitation facilities, and financial resources. Food safety standards can be implemented in developing nations to reduce the incidence of foodborne illnesses, improve the quality of life for the population, and facilitate export market access [2,11]. However, developed nations are hindered by complex regulatory environments and limited resources. [5,12,13,14,15,16].

Developing countries face challenges due to limited resources, weak regulations, and low awareness of food safety. However, capacity-building initiatives aim to address these challenges and improve food safety practices in developing nations [23].

- Prerequisite Programs

Implementing and complying with these fundamental PRP programs may pose challenges in developing countries due to limited resources, cultural factors, and lack of knowledge and awareness. [1,11,34].

- Traceability Programs

Developing countries face challenges in implementing traceability programs due to limited resources, inadequate infrastructure, low literacy rates, and a lack of automation. This results in difficulties in tracking and tracing food products, which can increase the risk of foodborne illnesses. [11,28].

- Food Safety Culture (FSC)

This study on the food safety culture element of FSMS and awareness in developing countries found that food safety culture is low due to low awareness and poor knowledge of food safety, inadequate infrastructure, and regulatory standards, challenges implementing food safety guidelines, and insufficient financial resources to develop a robust food safety culture. The significance of the elements of food safety culture lies within the human factor and specific training including parameters such as leadership, commitment, communication, risk awareness, work environment, and management system, styles, and processes [80,81,82]. A more recent work developed an assessment tool that can be used to assess food workers’ perceptions of their restaurant’s food safety culture [83]. The highest-rated construct was Resource Availability, i.e., the availability of resources to maintain good hand hygiene. The second highest-rated construct was Employee Commitment, which assessed workers’ perceptions of their coworkers’ commitment to food safety.

The last two constructs were related to management. Leadership assessed the existence of food safety policies, training, and information sharing. The Management Commitment assessed whether food safety was a priority in practice. Finally, they assessed Workers’ Beliefs about Food Safety Culture. We should not forget that food safety culture needs to be established and maintained according to The Commission Regulation (EU) No. 2021/382 (European Commission, 2021), amending the Regulation (EC) No. 852/2004 (European Commission, 2004). The Reg. (EU) No. 2021/382 did not detail methodologies for FSC implementation, investigation, or improvement, which have been recently evaluated by the European Commission Notice 2022/C 355/01 (European Commission, 2022). It contains an example of indicators for FSC assessment, to guide companies towards a successful FSC adoption and dissemination, and a checklist to evaluate FSC for official control activities [84].

### 4.5. Areas for Further Research 

A much more systematic approach needs to be adopted to investigate how the FSMS interacts with other variables, such as food safety and quality culture. It is also important to see the human factor involvement in these systems and how effective training and communication could help towards a better understanding and maturation of the systems employed by food firms. A strong culture always helps firms to make decisions and act fast on recalls, or customer complaints. Models, guidelines, assessment tools and customization examples need to be developed for FSC evaluation, as well as strategies for its dissemination within companies and along the supply chain. Of course, this requires effective management commitment and effective communication across all company levels.

## 5. Conclusions

Attitudes towards the constraints and benefits of implementing FSMS standards before and after implementation in developed and developing countries showed that after implementation there was a significant increase in the percentage of companies that have implemented the international FSMS both in developed (16.7% to 63.9%) and developing countries (26.6% to 48.1%). Certification has also increased from 34.2% to 59.6% in the total sample, namely from 33.3% to 61.1% in developed countries and from 34.6% to 59.0% in developing countries. Food safety culture and manager leadership implementation were increased to over 80% after FSMS implementation in both developed and developing countries (*p* < 0.001).

Key Performance Indicator (KPI), Multi-FSMS, Workers Training, Sustainability Programs, Food & Materials Waste, Reduction Programs, Lot Identification Traceability, Crisis Management, Food Defense TACCP Plan and Food Fraud VACCP Plan implementations also increased after FSMS implementation for all companies participating in the sample (*p* < 0.001). Attitudes towards food security in the region of the company’s activities were more positive after the implementation of FSMS in developing countries (53.1% to 78.1%, *p* < 0.001. There was a decrease in companies reporting that FSMS implementation is an expensive and complicated task, showing economic, technological, and legislation constraints (*p* < 0.001), while there was an increase in companies reporting the non-familiarity of FSMS to customers and consumers (*p* < 0.001).

Regarding differences in geographical regions, European companies reported significant increases in changes/upgrades of the FSMS standards, application of quality standards, GFSI implementations, food safety culture and manager leadership applications. North American companies (US and Canada) indicated a significant increase in the implementation of International FSMS, Certification and SQF integration, changes which were not detected in Europe. For Indian companies, international FSMS application and certification have presented a significant increase, larger than the ones reported for African or Latin American companies.

No significant change in the incentives to implement FSMS Standards was detected within different sectors before and after FSMS implementation. The sectors of processed fruit and vegetables, snacks and baked goods, food service, ingredients, and meat have reported greater constraints of non-familiarity of FSMS standards to consumers and customers after FSMS implementation.

According to the findings of the study, there are still gaps in the implementation of food safety standards, particularly in the areas of HACCP and microbiological standards. To ensure the effective implementation and enforcement of food safety standards, governments should allocate adequate resources and strengthen their regulatory frameworks. In this context, companies should improve food safety culture by empowering different dynamics with the aid of models, guidelines and assessment tools.

## Figures and Tables

**Table 1:** Examples of Micro-organisms, Parasite, Toxins, and Metabolites [6].

Category	Examples of Micro-Organisms, Parasites, Toxins and Metabolites
Raw meat: Carcasses of cattle, sheep, goats, pigs, and horses	*Salmonella* spp., *Escherichia coli*, *E.coli O157:H7*, *Yersinia enterocolitica*, *Campylobacter jejuni*, *Listeria monocytogenes*, *Clostridium botulinum* and *C. perfringens*, *Staphylococcus aureus* Parasites: *Toxoplasma*, *Trichinella*, *Taenia* and *Sarcocystis* Mycotoxins: Obtained from the animal through animal feed
Carcasses of broilers and turkeys	*Salmonella* spp., *Escherichia coli*, *Campylobacter* spp., *Listeria monocytogenes*, *Clostridium botulinum* and *C. perfringens*, *Staphylococcus aureus*, *Bacillus cereus*
Milk and dairy products	*Salmonella* spp., *Escherichia coli*, *E.coli O157:H7*, *Campylobacter* spp., *Listeria monocytogenes*, *Bacillus cereus*, *Staphylococcus aureus*, *Brucella* spp. Mycotoxins (aflatoxins Μ_1_ and Μ_2_), toxins from *S. aureus* and *C. botulinum* (mainly from yogurt containing fruits or nuts) shelfish
Egg products	*Enterobacteriaceae* (mainly *Salmonela* spp.), *L. monocytogenes*
Fishery products	*Staphylococcus* spp., *Clostridium botulinum, Vibrio* spp., *Vibrio parahaemolyticus*, histamine-producing bacteria (*Proteus morganii*), *Enterobacter* spp., *Citrobacter* spp., *Salmonella* spp., *Erysipelothrix rhusiopathiae* Parasites: *Diphyllobothrium latum*, *Clonorchis sinensis*, *Pseudoterranova* spp. Toxins: *Scombrotoxin, Ciguatera and Histamine*
shellfish	*Escherichia coli*, Toxins: *Amnesic shelfish toxin* (ASP), *Diarrhoetic shelfish toxin* (DSP), *Neurotoxic shellfish toxin* (NSP), *Paralytic shelfish toxin* (PSP), *Ciguatera*
Vegetables, fruits, and products thereof	*Escherichia coli, Salmonella* spp., *Shigella* spp., *Listeria* spp., *Staphylococcus* spp. (mainly in mushrooms) Toxins: *Mycotoxins* (Patulin mainly in apples and apple juice, pears and peaches), *aflatoxins* (mainly in figs), toxins from *Clostridium botulinum*
Cereals and nuts	*Salmonella* spp., *B. cereus*, *S. aureus* *Mycotoxins* (aflatoxins)
Milk powder & powdered infant formula, fruit, vegetables, cereals, starches, dry food & meat products	*Cronobacter* spp.

**Table 2:** Descriptives for company characteristics in the sample of this study.

	Total Sample (N = 116)		Developed (N = 36)		Developing (N = 80)	
	N	%	N	%	N	%
***Sectors***						
Agri	6	5.2	2	5.6	4	5.0
Beverage.Water	5	4.3	2	5.6	3	3.8
Canning/Bottling/Packaging	6	5.2	2	5.6	4	5.0
Coffee/Condiments/Sugar	8	6.9	0	0.0	8	10.0
Dairy	11	9.5	3	8.3	8	10.0
Services (Education, sanitary, transport, storage etc.)	7	6	2	5.6	5	6.3
Food service	11	9.5	6	16.7	5	6.3
Fruit.Veg-Processed	16	13.8	5	13.9	11	13.8
Ingredients	11	9.5	0	0.0	11	13.8
Meat	8	6.9	3	8.30	5	6.3
Snacks.BakedGoods	14	12.1	4	11.1	10	12.5
Other	13	11.2	7	19.4	6	7.5
***Countries***						
Africa (Algeria, Cameroon, Ethiopia, Ghana, Nigeria, Pakistan, South Africa)	24	20.7	0	0.0	24	30.0
UK and Australia	7	6.0	7	19.4	0	0.0
Latin America and the Caribbean (Brazil, Jamaica, Mexico, Trinidad)	16	13.8	0	0.0	16	20.0
North America (USA, Canada)	12	10.3	12	33.3	0	0.0
South Asia (India)	16	13.8	0	0.0	16	20.0
Other South Asia (Iran, Pakistan)	10	8.6	0	0.0	10	12.5
East and West Asia (Myanmar, Vietnam, China, Jordan, UAE)	17	14.7	7	19.4	10	12.5
Europe	14	12.1	10	27.8	4	5.0

**Table 3:** Cronbach alpha for all parameters tested.

	Cronbach’s Alpha	
	Before	After
**FSMS Element Details**	0.740	0.718
**Prerequisites**	0.712	0.827
**HACCP**	0.927	0.938
**Constraints**	0.559	0.503
**Incentives**	0.828	0.828

## Data Availability

Not applicable.

## Supplementary Materials

The following supporting information can be downloaded at: https://www.mdpi.com/article/10.3390/foods12173218/s1, Sample Case Study Survey [M1].

## Appendix A

Acronyms Glossary	
BRCGS	Brand Reputation Compliance Global Standard
CA	Corrective Action
CAR	Corrective Action Report
CL	Control Limit
CM	Control Measure
CCP	Critical Control Point
EMP	Environmental Monitoring Program
FDP	Food Defense Plan
FF	Food Fraud
FSMS	Safety Management System
FSC	Food Safety Culture
FSC	Food Sector Category
FSSC 22000	Food Safety System Certification
FSP	Food Safety Plan FSP
GAP	Good Agricultural Practices
GFSI	Global Food Safety Initiative
GMP	Good Manufacturing Practices
GHP	Good Hygiene Practices
GSP	Good Standardization Practices
HACCP	Hazard Analysis Critical Control Point
IFS	International Featured Standards
ISO 22000	International Standards Organization
Food Safety System	
oPRP	Operational Prerequisite Program
PDCA	Plan, Do, Check, Act
PRP	Prerequisite Program
RCA	Root Cause Analysis
SOP	Standard Operating Procedure
SQFI	Safe Quality Food Institute
SSOP	Sanitation Standard Operating Procedure
TACCP	Threat Analysis Critical Control Point
VACCP	Vulnerability Analysis Critical Control Point
