# Supplementary material for: Case Studies of Small-Medium Food Enterprises around the World: Major Constraints and Benefits from the Implementation of Food Safety Management Systems
Source: Foods. 2023 Aug 26;12(17):3218. doi: 10.3390/foods12173218 (PMC10486654; doi:10.3390/foods12173218)
Supplement: Supplementary file 1 [file foods-12-03218-s001.zip › foods-2554154-supplementary.pdf]

Sample Case Study Survey

CASE STUDY SURVEY # __3__ of _8_ Contributor: A.Berry email: _aberry@gmail.com_				
Company's Food Sector Category: Specialty FB Packaging				
Company's Site Location(s): region Northwestern country_USA___________				
Constraints and Benefits of Implementing FSMS Standards				
	**Case Study Questions**<br>**FSMS Element Details**	**BEFORE**<br>**Implementing**<br>**FSMS Standards**	**AFTER**<br>**Implementing FSMS Standards**	
		**Status**	**Benefits**	**Notes**
A.	Which FSMS Standards has your company implemented?<br>*(Your company FSMS profile may cover multiple check boxes)*	International<br>☐<br>National<br>☑ Mandatory<br>☑<br>Voluntary<br>☑<br>Certified<br>☐<br>Not Certified<br>☑	International<br>☑<br>National<br>☑ Mandatory<br>☑<br>Voluntary<br>☑<br>Certified<br>☑<br>Not Certified<br>☐	
B.	Name of current FSMS Standards<br>Or market based FSMS (HACCP/Preventive Control Based)<br>*If Other, please write name of Standards in Notes Column	ISO 22000<br>☐<br>BRC<br>☐ SQF<br>☑<br>IFS<br>☐<br>Food Standards Agency<br>☐<br>Other *<br>☑	ISO 22000:2018<br>☑<br>BRC<br>☐ SQF<br>☑<br>IFS<br>☐<br>Food Standards Agency<br>☐<br>Other*<br>☐	*Other<br>Industry Trade Standards
C.	When was your current FSMS Standards **first version** implemented?	2016-2022<br>☐<br>2010-2015<br>☑ 2005-2009<br>☐<br>2000-2008<br>☐<br>Pre 2000 ☐		
D.	Have you changed or upgraded your **FSMS Standards?**	☑ Yes ǀ ☐ No	☑ Yes ǀ ☐ No	
1	Any Food Safety Management System/Scheme before current **FSMS Standards?**	☑ Yes ǀ ☐ No	☑ Yes ǀ ☐ No	Industry Trade Standards
2	Quality Standards before and after **FSMS Standards?**	☑ Yes ǀ ☐ No	☑ Yes ǀ ☐ No	
2.1	Earlier version of **FSMS Standards** before?	☑ Yes ǀ ☐ No	☑ Yes ǀ ☐ No	

3	GFSI 3rd Party Certification before or after **FSMS Standards?**	☐ Yes | ☑ No	☑ Yes | ☐ No	Consultant Hired to conduct Gap Analysis
4	Non-GFSI 3rd Party Certification Scheme?	☑ Yes | ☐ No	☑ Yes | ☐ No	Industry Trade Standards
5	Regulatory Agencies Inspections: Local, State, Federal	Local ☑<br>State ☑<br>Federal ☑	Local ☑<br>State ☑<br>Federal ☑	
6	Small (**employees: 50 or less**) or Medium Size (**employees: 51-250**)	Small ☑<br>Medium ☐	Small ☑<br>Medium ☐	
7	Food Safety Culture	Implemented ☐<br>Not Implemented ☑	Implemented ☑<br>Not Implemented ☐	
8	Management Leadership	Implemented ☐<br>Not Implemented ☑	Implemented ☑<br>Not Implemented ☐	
9	Training/Awareness	Adequate ☐<br>Inadequate ☑	Adequate ☑<br>Inadequate ☐	Consultant hired to initiate in-house training
10	Resources	Adequate ☑<br>Inadequate ☐	Adequate ☑<br>Inadequate ☐	
11	Technology	Adequate ☑<br>Inadequate ☐	Adequate ☑<br>Inadequate ☐	AI Programs not cost effective
12	Production Yield	Adequate ☐<br>Inadequate ☑	Improved ☑<br>Same ☐	
13	Key Performance Indicator KPI	Implemented ☑<br>Not Implemented ☐	Implemented ☑<br>Not Implemented ☐	Need to hire skilled staff
14	Geographic Infra-Structure	Adequate ☑<br>Inadequate ☐	Adequate ☑<br>Inadequate ☐	
15	Multi-FSMS in Company	☑ Yes | ☐ No	☑ Yes | ☐ No	Eliminated non-essentials
16	Workers Training	Implemented ☐<br>Not Implemented ☑	Implemented ☑<br>Not Implemented ☐	Trying to handle labor shortage and turnovers
17	Sustainability Programs	Implemented ☑<br>Not Implemented ☐	Implemented ☑<br>Not Implemented ☐	
18	Food & Materials Waste Reduction Programs	Implemented ☐<br>Not Implemented ☑	Implemented ☑<br>Not Implemented ☐	
19	Lot Identification Traceability	Implemented ☑<br>Not Implemented ☐	Implemented ☑<br>Not Implemented ☐	
20	Crisis Management	Implemented ☐<br>Not Implemented ☑	Implemented ☑<br>Not Implemented ☐	
21	Food Defense TACCP Plan	Implemented ☐<br>Not Implemented ☑	Implemented ☑<br>Not Implemented ☐	
22	Food Fraud VACCP Plan	Implemented ☐<br>Not Implemented ☑	Implemented ☑<br>Not Implemented ☐	We are low risk for Food Fraud
23	Foodborne Illness in region **Select TOP 3**	*Campylobacter* ☑<br>*Clostridium perfringens*<br>☐ *E. coli*<br>☐<br>*Listeria monocytogenes*<br>☐<br>*Norovirus*<br>☑	*Campylobacter* ☑<br>*Clostridium perfringens*<br>☐ *E. coli*<br>☐<br>*Listeria monocytogenes*<br>☐<br>*Norovirus*<br>☑	

		Salmonella ☑<br>*Staphylococcus aureus* ☑	*Salmonella* ☑<br>*Staphylococcus aureus* ☐	
24	Food Security in Region	Adequate ☑<br>Inadequate ☐	Adequate ☑<br>Inadequate ☐	
	**Prerequisites**			Consultant Hired to Develop
25	Good Manufacturing Practices GMP Program	Practiced GMPs<br>☑ Yes I ☐ No<br>Program<br>Implemented ☐<br>Not Implemented ☑	Program<br>Implemented ☑<br>Not Implemented ☐	Practiced but no programs in place until FSMS Standards implemented
26	Good Hygiene Practices GHP Program	Practiced GHPs<br>☑ Yes I ☐ No<br>Program<br>Implemented ☐<br>Not Implemented ☑	Program<br>Implemented ☑<br>Not Implemented ☐	
27	Good Process Practices GPP Program	Practiced GPPs<br>☑ Yes I ☐ No<br>Program<br>Implemented ☐<br>Not Implemented ☑	Program<br>Implemented ☑<br>Not Implemented ☐	
28	Sanitation Standard Operations Procedures SSOP Program	Practiced SSOPs<br>☑ Yes I ☐ No<br>Program<br>Implemented ☐<br>Not Implemented ☑	Program<br>Implemented ☑<br>Not Implemented ☐	
29	Site Design	Conducted Site Design<br>☑ Yes I ☐ No<br>Program<br>Implemented ☐<br>Not Implemented ☑	Program<br>Implemented ☑<br>Not Implemented ☐	
30	Equipment Design	Conducted Equip Design ☑ Yes I<br>☐ No<br>Program<br>Implemented ☐<br>Not Implemented ☑	Program<br>Implemented ☑<br>Not Implemented ☐	
31	Internal Audits: frequency	None ☐<br>Annual ☑<br>Bi-Annual ☐<br>Quarterly ☐<br>Monthly ☐	None ☐<br>Annual ☐<br>Bi-Annual ☐<br>Quarterly ☑<br>Monthly ☐	

	HACCP			
32	Control Measures	Implemented ☑<br>Not Implemented ☐	Implemented ☑<br>Not Implemented ☐	
33	Control Points	Implemented ☐<br>Not Implemented ☑	Implemented ☑<br>Not Implemented ☐	
34	Operational Prerequisite Programs oPRPs	Implemented ☐<br>Not Implemented ☑	Implemented ☑<br>Not Implemented ☐	
35	Critical Control Points	Implemented ☐<br>Not Implemented ☑	Implemented ☑<br>Not Implemented ☐	
36	Processed Based Microbiological Criteria and Testing Practices	Implemented ☐<br>Not Implemented ☑	Implemented ☑<br>Not Implemented ☐	
37	Monitor, Verify, Validate, Record Keeping, Responsible Person in Charge PIC	Implemented ☐<br>Not Implemented ☑	Implemented ☑<br>Not Implemented ☐	
38	Deviations, Corrective Actions	Implemented ☐<br>Not Implemented ☑	Implemented ☑<br>Not Implemented ☐	
	**Constraints to Implement FSMS Standards (1=strongly disagree: 5=strongly agree)**			
1	Expensive and complicated task (i.e., there are economic, technological, and legislation constraints)<br>☐ 1 ☐ 2 ☐ 3 ☑ 4 ☐ 5		Lack of complete, accurate, timely, and easily accessible information about the need for FSMS<br>☐ 1 ☑ 2 ☐ 3 ☐ 4 ☐ 5	
	Strongly disagree	Strongly agree	Strongly disagree	Strongly agree
2	Resource-intensive, require much administration and paper works which place a burden on companies<br>☐ 1 ☐ 2 ☐ 3 ☑ 4 ☐ 5		Lack of trained staff for technical and management aspects of FSMS<br>☐ 1 ☐ 2 ☐ 3 ☑ 4 ☐ 5	
	Strongly disagree	Strongly agree	Strongly disagree	Strongly agree
3	Lack of clarity about the benefits to be gained from implementing FSMS vis-à-vis required investment costs<br>☐ 1 ☐ 2 ☐ 3 ☑ 4 ☐ 5		Not required by (non) governmental agencies<br>☐ 1 ☐ 2 ☐ 3 ☐ 4 ☑ 5	
	Strongly disagree	Strongly agree	Strongly disagree	Strongly agree
4	Not familiar to customers and consumers<br>☑ 1 ☐ 2 ☐ 3 ☐ 4 ☐ 5		Positive Food Safety Culture<br>Reduces risk and collectively empowers personnel<br>☐ 1 ☐ 2 ☐ 3 ☐ 4 ☑ 5	
	Strongly disagree	Strongly agree	Strongly disagree	Strongly agree
	**Incentives to implement FSMS Standards (1=strongly disagree: 5=strongly agree)**			

5	Reduces product losses<br>☐ 1 ☐ 2 ☐ 3 ☑ 4 ☐ 5		Streamlines paperwork<br>☐ 1 ☐ 2 ☐ 3 ☑ 4 ☐ 5	
	Strongly disagree	Strongly agree	Strongly disagree	Strongly agree
6	Avoids duplication between processes<br>☐ 1 ☐ 2 ☐ 3 ☑ 4 ☐ 5		Increases operational efficiency<br>☐ 1 ☐ 2 ☐ 3 ☐ 4 ☑ 5	
	Strongly disagree	Strongly agree	Strongly disagree	Strongly agree
7	Improves quality of management<br>☐ 1 ☐ 2 ☐ 3 ☐ 4 ☑ 5		Enhances export competitiveness<br>☐ 1 ☐ 2 ☐ 3 ☑ 4 ☐ 5	
	Strongly disagree	Strongly agree	Strongly disagree	Strongly agree
8	Enhances export competitiveness<br>☐ 1 ☐ 2 ☐ 3 ☑ 4 ☐ 5		Improves market share<br>☐ 1 ☐ 2 ☐ 3 ☑ 4 ☐ 5	
	Strongly disagree	Strongly agree	Strongly disagree	Strongly agree
9	Accesses to new markets<br>☐ 1 ☐ 2 ☐ 3 ☑ 4 ☐ 5		Provides competitive advantage<br>☐ 1 ☐ 2 ☐ 3 ☑ 4 ☐ 5	
	Strongly disagree	Strongly agree	Strongly disagree	Strongly agree
10	Improves company image<br>☐ 1 ☐ 2 ☐ 3 ☑ 4 ☐ 5		Reduces legal liability<br>☐ 1 ☐ 2 ☐ 3 ☑ 4 ☐ 5	
	Strongly disagree	Strongly agree	Strongly disagree	Strongly agree
11	Meets customers' requirements<br>☐ 1 ☐ 2 ☐ 3 ☐ 4 ☑ 5		Reduces risk of product recalls<br>☐ 1 ☐ 2 ☐ 3 ☑ 4 ☐ 5	
	Strongly disagree	Strongly agree	Strongly disagree	Strongly agree
12	Provides evidence of legal compliance<br>☐ 1 ☐ 2 ☐ 3 ☐ 4 ☑ 5		Provides customer assurance<br>☐ 1 ☐ 2 ☐ 3 ☐ 4 ☑ 5	
	Strongly disagree	Strongly agree	Strongly disagree	Strongly agree

References:

1.Carmen Escanciano, Maria Leticia Santos-Vijande; Reasons and constraints to implementing an ISO 22000 food safety management system: Evidence from Spain; Elsevier, Food Control 40 (2014) 50-57; 19 November 2013 [63].

2. Gumataw Kifle Abaebe, Rachel Anne Bahn, Ali Chalak, Abed Al Kareem Yehya; Drivers for the implementation of market-based food safety management system: Evidence from Lebanon; Wiley, Food Science & Nutrition DOI: 10.1002/fsn31394; 13 December 2019 [64].

Dear Case Study Contributor,

Thank you for your valued time to complete the Constraints and Benefits from Implementation of Food Safety Management Systems Standards survey.

Disclosure:

Your case study contribution(s) data will be included within a collective statistical analysis to be part of our research paper "Major Constraints and Benefits from Implementing Food Safety Management Systems Standards for Small to Medium Sized Enterprises Around the Globe: Case Studies from Different Companies".

Confidentiality will be maintained. Therefore, this survey/questionnaire does not ask for company name, city, etc.

We ask non-specific general questions to gather pertinent information to be able to analyze and present the collective data in an informative format.

"The aim of the research paper is to evaluate the young history of Food Safety Management Systems Standards and its major constraints and benefits arising from its implementation by small to medium size enterprises around the world.

How to better improve or alleviate the constraints and to catapult the success of world food safety best management practices programs, HACCP and food safety management systems. Finally, to arrive at a consensus as to whether the implementation of Food Safety Management Systems Standards in small to medium food enterprises has so far impacted global foodborne illness (for better or worse)."
